# The developing pig respiratory microbiome harbors strains antagonistic to common respiratory pathogens

**DOI:** 10.1128/msystems.00626-24

**Published:** 2024-09-17

**Authors:** Abel A. Vlasblom, Birgitta Duim, Shriram Patel, Roosmarijn E. C. Luiken, Daniel Crespo-Piazuelo, Julia Eckenberger, Chloe E. Huseyin, Peadar G. Lawlor, Christian Elend, Jaap A. Wagenaar, Marcus J. Claesson, Aldert L. Zomer

**Affiliations:** 1 Faculty of Veterinary Medicine, Division of Infectious Diseases and Immunology, Utrecht University, Utrecht, The Netherlands; 2 WHO Collaborating Centre for Reference and Research on Campylobacter and Antimicrobial Resistance from a One Health Perspective/WOAH Reference Laboratory for Campylobacteriosis, Utrecht, The Netherlands; 3 School of Microbiology and APC Microbiome Ireland, University College Cork, Cork, Ireland; 4 SeqBiome Ltd., Cork, Ireland; 5 Pig Development Department,Teagasc Animal & Grassland Research & Innovation Centre, Moorepark, Fermoy, Co. Cork, Ireland; 6 EW Nutrition Innovation GmbH & Co.KG, Cologne, Germany; 7 Wageningen Bioveterinary Research, Lelystad, The Netherlands; California State University Stanislaus, Turlock, California, USA

**Keywords:** microbiome, respiratory pathogens, colonization, porcine, nasal, microbiome development

## Abstract

In the global efforts to combat antimicrobial resistance and reduce antimicrobial use in pig production, there is a continuous search for methods to prevent and/or treat infections. Within this scope, we explored the relationship between the developing piglet nasal microbiome and (zoonotic) bacterial pathogens from birth until 10 weeks of life. The nasal microbiome of 54 pigs was longitudinally studied over 16 timepoints on 9 farms in 3 European countries (Germany, Ireland, and the Netherlands) using amplicon sequencing targeting the V3-V4 16S rRNA region as well as the *tuf* gene for its staphylococcal discrimination power. The piglets’ age, the farm, and the litter affected the nasal microbiome, with piglets’ age explaining 19% of the variation in microbial composition between samples. Stabilization of the microbiome occurred around 2 weeks post-weaning. Notably, while opportunistic pathogens were ubiquitously present, they did not cause disease. The piglet nasal microbiome often carried species associated with gut, skin, or vagina, which suggests that contact with the vaginal and fecal microbiomes shapes the piglet nasal microbiome. We identified bacterial co-abundance groups of species that were present in the nasal microbiomes in all three countries over time. Anti-correlation between these species and known bacterial pathogens identified species that might be exploited for pathogen reduction. Further experimental evidence is required to confirm these findings. Overall, this study advances our understanding of the piglet nasal microbiome, the factors influencing it, and its longitudinal development, providing insights into its role in health and disease.

**IMPORTANCE:**

Our study on the nasal microbiota development in piglets across farms in three European countries found that the microbiomes developed similarly in all locations. Additionally, we observed that the colonization of porcine pathogens was either positively or negatively associated with the presence of other bacterial species. These findings enhance our knowledge of co-colonizing species in the nasal cavity and the identified microbial interactions that can be explored for the development of interventions to control pathogens in porcine husbandry.

## INTRODUCTION

In pig farming, bacterial respiratory and systemic infections can be detrimental to health and welfare, and increase cost and antimicrobial use (1, 2). There is a continuous search for (biological) interventions, such as probiotics and competitive exclusion strategies, to prevent and treat infections (3–5).

The impact of the respiratory microbiome on piglet respiratory or systemic infections is an emerging field (6–10). Differences in (upper) airway microbiome composition between healthy and diseased pigs (9–15), between livestock-associated methicillin-resistant *Staphylococcus aureus* (LA-MRSA) carriers and non-carriers (7, 16, 17) and due to exposure to farm conditions, such as high gaseous ammonia concentrations (18), have been described, often in cross-sectional studies. These studies have identified species associated with disease. Longitudinal piglet nasal studies focusing on microbiome development have been performed (7, 19, 20). However, longitudinal development of the pig nasal microbiome (PNM) in relationship with bacterial pathogens is understudied. By elucidating the PNM’s intricacies, we will obtain insights that might improve animal health or reduce the presence of bacterial species with zoonotic potential through microbiome modulation.

Common opportunistic bacterial pathogens, including those part of the porcine respiratory disease complex (PRDC) (1, 21), such as *Actinobacillus pleuropneumoniae* (22), *Actinobacillus suis* (23), *Trueperella pyogenes* (24), *Bordetella bronchiseptica* (22), *Glaesserella parasuis* (9), *Klebsiella pneumoniae* (25), *Mannheimia varigena* (26), *Mycoplasma hyopneumoniae* (22), *Mycoplasma hyorhinis* (27), *Pasteurella multocida* (28), and *Streptococcus suis* (29), are often present in the porcine upper respiratory tract (30). In addition, potential zoonotic pathogens such as LA-MRSA reside in the respiratory tract.

To elucidate trends in the development of the PNM, including its relationship with the relative abundance of these 12 pathogens, 54 piglets were sampled (nasal swabs) across 9 farms in 3 European countries from birth until 70 days of age for *tuf* and 16S rRNA gene amplicon sequencing. *tuf* Amplicon sequencing improved taxonomical resolution, over 16S rRNA sequencing, for some clinically relevant species, including *Staphylococcus*, *Enterococcus*, and *Streptococcus* (7, 31, 32). Member of these genera might be of interest because they can interfere with nasal colonization of *S*. *aureus* (33). Correlation network analysis (clustering based on positive and negative associations in species abundance) identified co-abundance groups (CAGs) of co-occurring bacterial species, and singular species, which displayed anti- and co-correlation to the selected bacterial pathogens.

## RESULTS

### Cohort characteristic and sampling summary

Nasal swabs were obtained from 54 piglets born to 27 sows across 9 farms equally distributed in 3 countries (Table 1). We sampled from birth up to 10 weeks of age; daily during the first week of life, as our previous work showed rapid microbiome developed during this period (7), and weekly thereafter. Piglets were weaned between timepoints 26 and 31 with most farms weaning was performed at timepoint 28. The 16S rRNA was sequenced from all samples (*n* = 813; Table 1). Furthermore, samples from two of the three litters per farm were additionally *tuf* amplicon sequenced (*n* = 538) for improved *Staphylococcus* resolution (31). Due to sequencing failure, 21 samples were lost (dark gray values in Table 1). Another 30 samples were excluded due to doxycycline treatment after timepoint 27 in farm NLD3 (light gray 0 values in Table 1).

**TABLE 1:** Distribution of nasal swab samples used for amplicon sequencing^*d*^

				Timepoints																
Country	Farm ID	Litters	Piglets	0	1	2	3	4	5	6	13	20	27	34	41	48	55	62	69	Total samples
Germany	GER1	3	6	6 ^*a*^	6	6	6	6	6	6	6	6	6	6	6	6	6	6	5	95
	GER2	3	6	6	6	6	6	6	6	6	6	6	6	6	6	6	6	6	5	95
	GER3	3	6	6	6	6	6	6	6	6	6	6	6	5	6	5	6	6	6	94
Ireland	IRL1	3	6	6	6	6	6	6	6	6	6	6	6	6	6	6	6	6	6	96
	IRL2	3	6	6	6	6	6	6	6	6	6	6	6	6	6	6	6	6	6	96
	IRL3	3	6	6	6	6	6	6	6	6	6	6	6	6	6	6	6	6	6	96
The Netherlands	NLD1	3	6	6	6	6	6	6	6	5	5	6	6	6	6	6	6	6	6	94
	NLD2	3	6	4	4	6	6	6	6	4	6	6	6	6	6	6	6	6	6	90
	NLD3	3	6	4	6	6	6	6	5	6	6	6	6	0	0	0	0	0	0	57
**Total**	**9**	**27**	**54**	50^*b*^	52	54	54	54	53	51	53	54	54	47	48	47	48	48	46	**813^*c*^**

^*a*^ The total number of nasal swabs collected per farm per timepoint. Timepoints are depicted in days (0–69) and represent the piglet’s age.

^*b*^ Total number of nasal swabs per timepoint.

^*c*^ Total number of included nasal swabs.

^*d*^ Dark grey values represent timepoints with sample loss.

### Summary of the sequencing results

Sequencing generated 32 and 15 million raw 16S rRNA and *tuf* gene reads, respectively. Microbiome analysis was performed on 17 and 11 million curated reads with a mean count of 19,812 ± 5,298 standard deviation (SD) and 18,226 ± 5,782 SD reads/sample for 16S rRNA and *tuf*, respectively (Fig. S1A and B; Fig. S2A and B). For 16S rRNA, 11 negative controls had a mean read count of 1,204 with 3 of the 11 negative controls containing ~4,000 reads. For *tuf*, seven negative controls were sequenced, with each of them having very few reads in them (<20 reads). Overall, 10,088 unique amplicon sequence variants (ASVs) were identified in the 16S rRNA data set, 1,430 of which were detected as potential contaminants. For *tuf*, 4,038 unique ASVs were generated with no potential contaminants. In the 16S rRNA data set, the phylum *Proteobacteria*, with a 65.4% mean relative abundance, was most prevalent followed by *Firmicutes* (20.8%) and *Bacteroidetes* (7.2%). At genus level, *Moraxella* (44.9%) had the highest mean relative abundance followed by *Streptococcus*, *Mannheimia, Rothia*, and *Actinobacillus* all at ~5%. For *tuf*, *Proteobacteria* and *Firmicutes* made up around 100% (59.5% and 39.7%, respectively) of the detected phyla, and the most relatively abundant genera were *Moraxella* (59.0%) and *Streptococcus* (16.8%).

### Bacterial diversity changes in the porcine nasal microbiome over time

**Fig 1:**
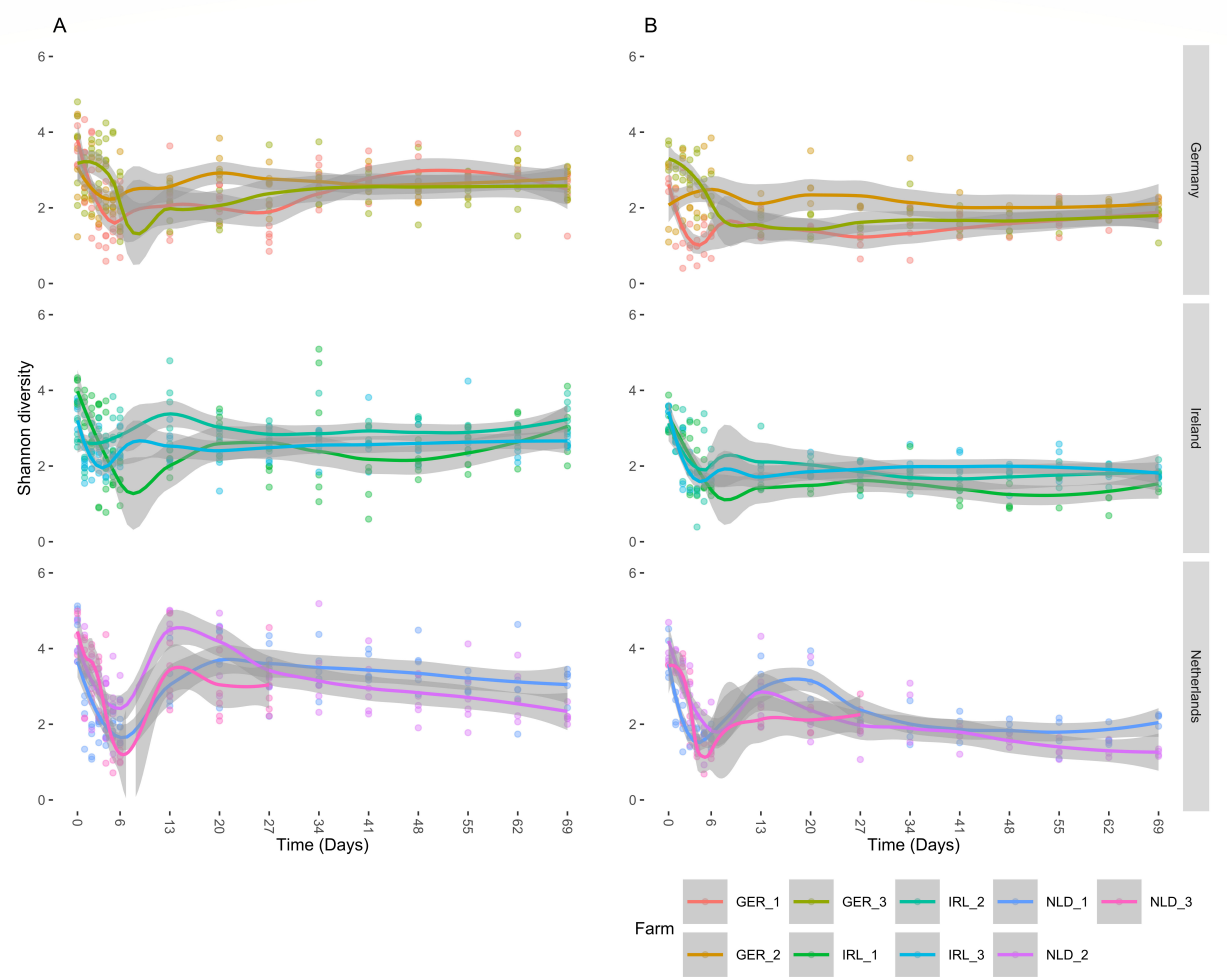
Longitudinal changes in the piglet nasal community Shannon diversity measured for (**A**) 16S rRNA and (**B**) *tuf* gene amplicon sequencing. X-axis: Sample time (days), timepoint 0 represents the piglets’ day of birth. Y-axis: Shannon diversity index. Farms are presented by country and color coded for identification. The dots represent the samples, lines display the predicted mean, and the gray boundaries the standard error.

The species diversity estimated with the Shannon index was higher at birth (Fig. 1; Fig. S3 with a categorical x-axis) for the *tuf* and 16S rRNA data sets with a lower overall richness for *tuf*. We observed a diversity decrease in the first week of life (T = 0 vs 6, *P* < 0.00001), which was most pronounced in the Netherlands, followed by a diversity increase at T = 13 (*P* < 0.00001). In the 16S rRNA data set, there was no significant difference in alpha diversity between timepoints 13 and 20, and 34 and 41, spanning over the time of weaning (T = 26–31). For *tuf,* there was also an alpha diversity decrease between the timepoints 0 and 6 (T = 0–6, *P* < 0.00001). However, between T6 and T13, no significant increase in alpha diversity was observed. We found no significant change in average Shannon diversity between T13 or T20, and T34 but did find a difference in Shannon diversity of weeks 2 and 3 (T = 13 and 20) vs week 6 (T = 41; *P* 0.015 and *P* 0.003), with week 6 samples having lower average Shannon diversity than the earlier samples. Between week 5 (T = 34) and week 10 (T = 69), we did not observe a significant difference in alpha diversity in both data sets.

### Beta diversity displays country-independent microbiome development

**Fig 2:**
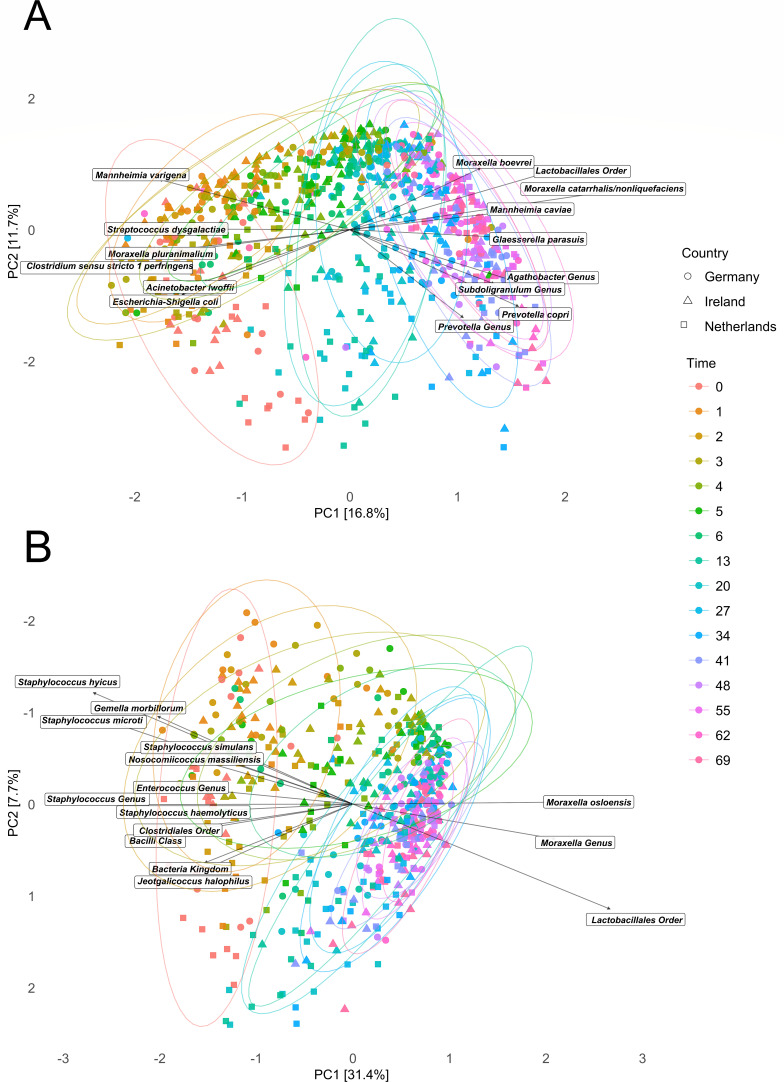
Principal component analyses show compositional differences and similarities (beta diversity) in the nasal microbiomes of piglets over time in the 16S rRNA data (**A**) and the *tuf* data (**B**). Samples were colored by age of the piglets in days (0–69) and given shape by country (Germany, Ireland, and the Netherlands). The arrows show the top 15 taxa that drive the sample ordination. ^*^ represents a significant *P*-value (α < 0.05).

To investigate compositional changes in the piglet nasal microbiota over time, beta diversity was assessed via principal component analysis (PCA) based on Aitchison distances (Fig. 2). We observed across all three countries, an effect of time. Time (age of the piglet) explained most of the variation, as validated by permutational analysis of variance (PERMANOVA) in both the 16S rRNA and *tuf* data sets (19.24% and 24.73%, respectively; Table 2). Samples taken at birth (T = 0) are distinctly clustered from samples taken at the end of week 1 (T = 6) and week 5 (T = 34) (pairwise PERMANOVA, 16 S *R*^2^: 14%, 18%; *tuf R*^2^: 25%, 28%; *P* 0.001). Between week 3 (T = 20) and week 5 (T = 34) (spanning weaning), we observed a significant difference in beta diversity (pairwise PERMANOVA, *R*^2^: 7.2% and 6.7%; *P* 0.001 and 0.001; 16S and *tuf*). Late samples, between week 5 (T = 34) and week 10 (T = 69), appear overlapping/similar in the PCA; however, pairwise PERMANOVA found a significant difference between the timepoints in the 16S and *tuf* data sets (T = 34 vs 69; *R*^2^: 3.5% and 3.4%, *P*: 0.001 and 0.025) albeit with the smallest *R*^2^ values compared to other timepoint comparisons.

**TABLE 2:** PERMANOVA outputs for the 16S rRNA and *tuf* data sets with time, sex, and the nested factor country, farm, sow, piglet as factors explaining variation

16S rRNA					*Tuf*			
Factor	Df	SumOfSqs	*R* ^2^	Pr (>*F*)	Df	SumOfSqs	*R* ^2^	Pr (>*F*)
Time	15	132473.46	0.1924	0.0002^*a*^	15	29408.61	0.2473	0.0002^*a*^
Sex	1	1794.97	0.0026	0.0008^*a*^	1	462.00	0.0039	0.0030^*a*^
Country	2	19065.64	0.0277	0.0002^*a*^	2	3972.26	0.0334	0.0002^*a*^
Country: farm	6	36632.69	0.0532	0.0002^*a*^	6	8678.21	0.0730	0.0002^*a*^
Country: farm: sow: piglet	18	25908.57	0.0376	0.0002^*a*^	9	4159.90	0.0350	0.0002^*a*^
Country: farm: sow: piglet	26	14908.61	0.0216	0.8660	17	2122.51	0.0178	0.8676
Residual	744	457918.93	0.6649	NA^*b*^	487	70125.58	0.5896	NA
Total	812	688702.86	1	NA	537	118929.1	1	NA

^*a*^ Represents a significant adjusted *P*-value (α <0.05).

^*b*^ NA,Not applicable.

To indicate ordination-driving species, we plotted their effect as arrows in the 16S rRNA PCA. Earlier samples were driven by members of the genera *Clostridium*, *Moraxella*, *Streptococcus, Acinetobacter*, and *Escherichia*, where late samples were driven by members of the genera *Moraxella*, *Glaesserella*, *Agathobacter*, *Subdoligranulum*, *Prevotella*, and *Mannheimia* (Fig. 2A). For *tuf,* 12 of the top 15 ordination driving taxa were attributed to samples from earlier timepoints. *Moraxella osloensis*, *Moraxella* genus, and the Lactobacillales order drove the shift toward later timepoint clusters (Fig. 2B).

Additionally, we sought to identify factors that explained most of the variation in the beta diversity, apart from the factor time (time: 19.24% 16S and 24.73% *tuf*). We therefore tested the explained variance of the hierarchical levels and found that all levels explained some of the variance, but farm explained a small majority (5.3% 16S and 7.3% *tuf*; Table 2). Sex of piglets had a significant effect but explained, 0.26% for 16S and 0.39% for *tuf* of the variation (Table 2).

### Temporal trends in relative abundances at species-level taxonomy

**Fig 3:**
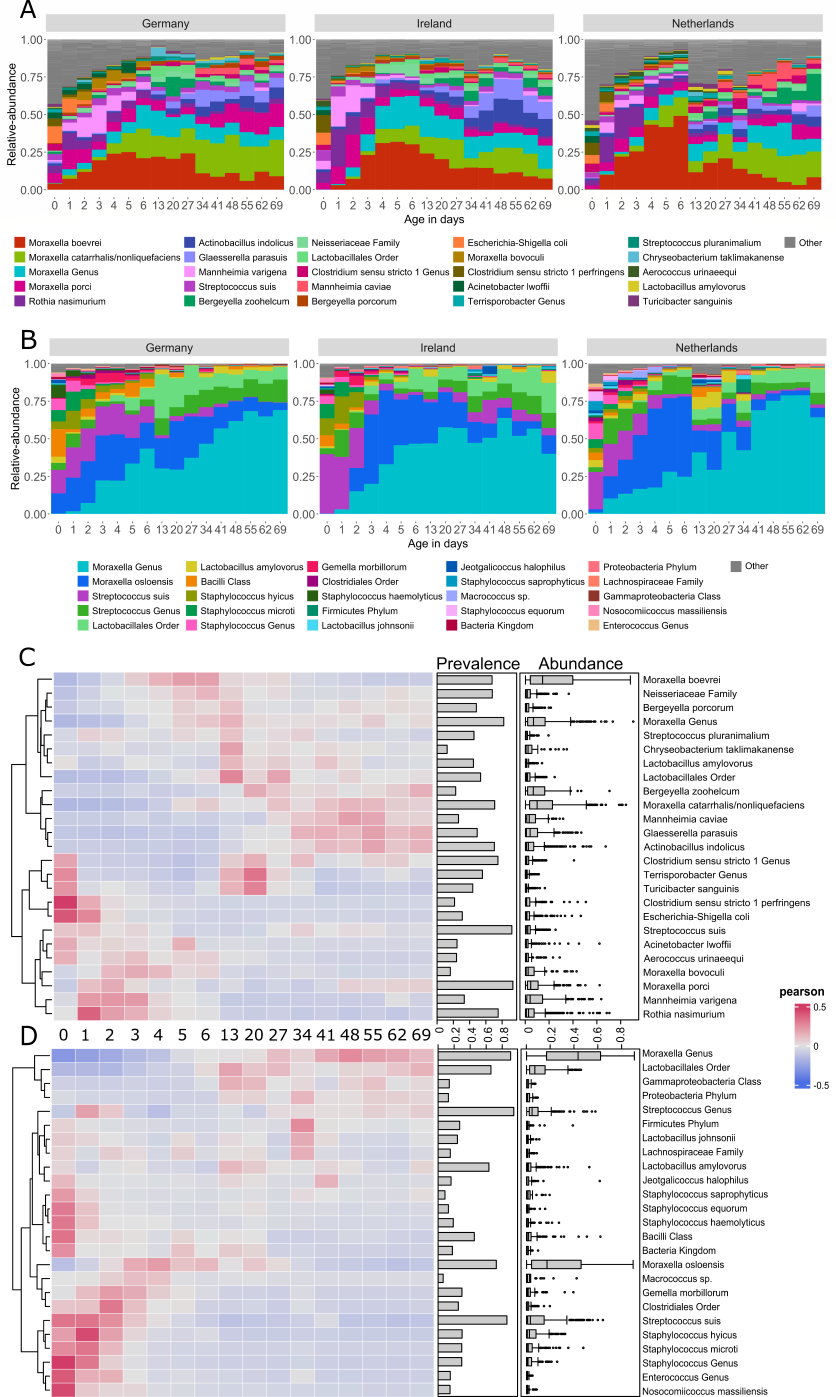
The top 25 most abundant taxa at species level in the 16S rRNA (**A**) and *tuf* (**B**) data in all samples per country. The piglet samples are grouped by age of the piglet in days. The heatmaps show, for each of the 25 top species, at which timepoint each species had its presence in the 16S rRNA (**C**) and *tuf* data (**D**); red correlates with a high presence, and blue correlates with a low presence of a taxon per timepoint (based on Pearson correlation). The bars to the right of the heatmap show the species prevalence over all samples, and boxplots show the distribution of its relative abundance over all samples.

Individual farm-level differences were observed among the top 25 most abundant taxa at species level (Fig. S4). However, trends in average relative abundances of the PNM top 25 species between the countries seemed similar (Fig. 3). For 16S rRNA, the most abundant species were members of the genus *Moraxella,* which often accounted for half of the species relative abundance. During the first week after birth, *M. boevrei,* together with *M. bovoculi* and *M. porci,* were the most abundant. Toward the end of the first week, the species *M. catarrhalis/nonliquefaciens* and an unknown species of the genus *Moraxella* became more prevalent. The species *M. porci* was consistently observed throughout all timepoints (prevalence bar, Fig. 3C), with its highest relative abundance beginning at week 1 and after day 48 with a pronounced larger proportion in the later German samples (Fig. 3A). *Streptococcus suis* was prevalent throughout with a low mean relative abundance. During the first week, we prevalently observed the species *Rothia nasimurium* and *Mannheimia varigena*, and species from the genera *Clostridium* and *Escherichia* were abundant. From the end of week 1 to week 10, we observed an increasing fraction of *Actinobacillus indolicus* (most pronounced in Ireland)*, Bergeyella zoohelcum,* and *Glaesserella parasuis*. These species together with the various *Moraxella* species comprised on average half of the averaged relative abundances in weeks 1–10. In the Dutch samples, in week 2 (T = 13), fewer of the top 25 most abundant species were present (Fig. 3A and B). This was clearly observed in Dutch farm NLD2 (Fig. S4A).

A lower number of taxa were observed for *tuf* (as compared to 16S), an amplicon known for a good resolution for *Staphylococcus*, *Streptococcus*, and *Enterococcus* species (31, 34, 35). In the *tuf* data set, the *Moraxella* genus was by far the most abundant taxon, specifically an unclassified member of the genus *Moraxella* (Fig. 3B). The second most relatively abundant *Moraxella* species was *M. osloensis,* which could not be differentiated by 16S rRNA gene sequencing and was abundant until weaning (T = 27). The species *S. suis* had its highest relative abundance in the first days of life but was detected throughout the sampling period shown in Fig. 3B and by the high prevalence bar in Fig. 3D. Around week 2, the *Streptococcus* genus and Lactobacilliales order, both without better taxonomic resolution, became more abundant.

Like 16S rRNA, in the Dutch *tuf* samples, the top 25 species explained less relative abundance in week 2 (T = 13). Overall, the top 25 taxa at species level comprised 90%–100% of the average relative abundance. After timepoint week 3 (T = 20), four to five species made up 90% of the relative abundance, illustrating the low number of taxa represented by *tuf*.

### Temporal trends of bacterial pathogens in the piglet nasal microbiota

**Fig 4:**
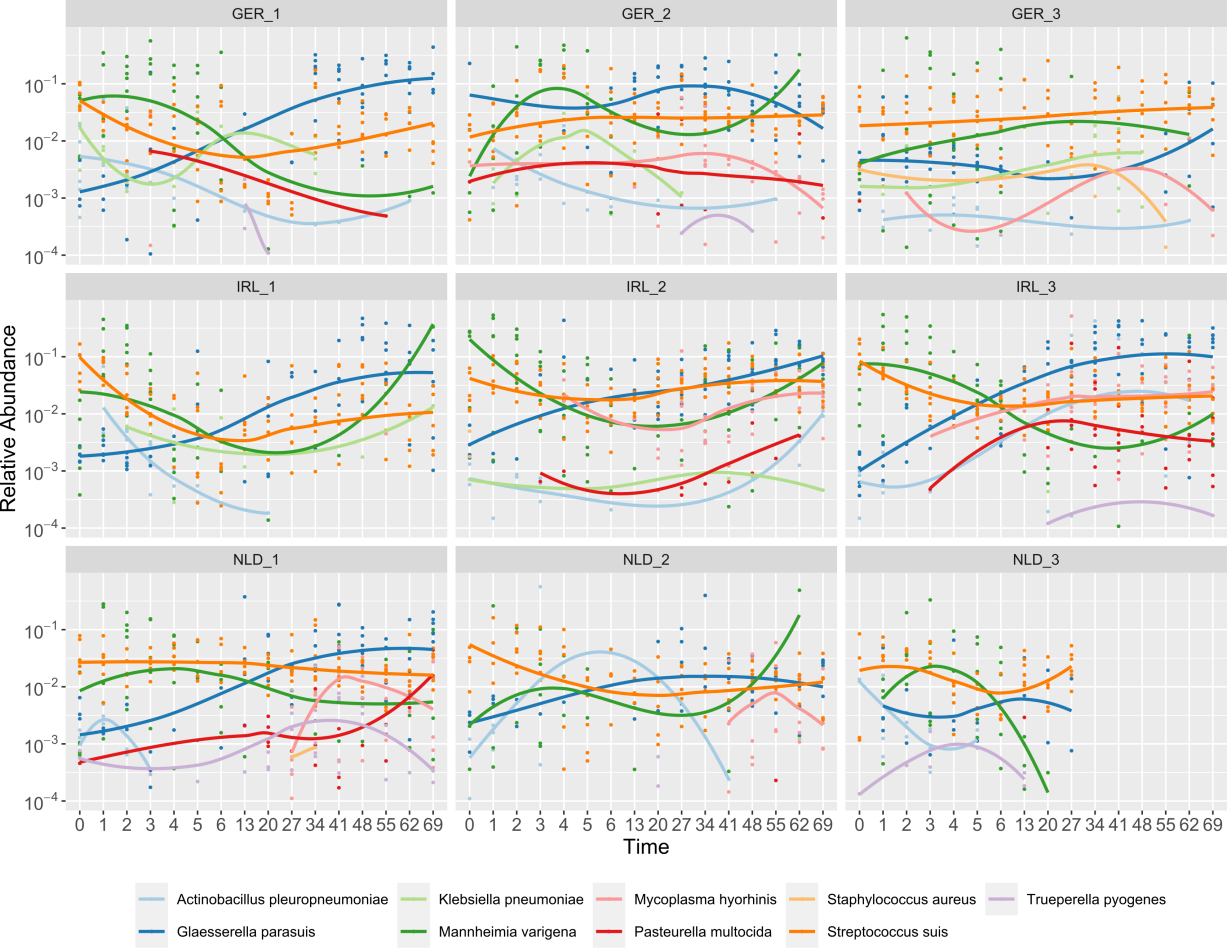
Log-transformed relative abundances (>0.0001) for 16S rRNA over time of the nine detected pig pathogens faceted per farm. Lines indicate trends in relative abundance within a farm.

Next, we focused on the 12 pig pathogens as in the Introduction. The relative abundances of these species were calculated for 16S and *tuf*, specified in Table 3. Of the 12 species, 9 were detected using 16S rRNA and 2 using *tuf* (*S. aureus* and *S. suis*). In the 813 samples, *S. suis* was the most prevalent (804/813) followed by *G. parasuis* (500/813) and *M. varigena* (365/813). These species also had the highest mean relative abundance of the 12 putatively pathogenic species, around 3% across samples. *M. varigena* had the highest mean relative abundance of 7.5%. The maximum relative abundance of *M. varigena, A. pleuropneumoniae*, *G. parasuis*, and *M. hyorhinis* was around 50%, and *S. aureus* had the lowest maximum relative abundance at around 0.7%. The species *Bordetella bronchiseptica* was not identified, but ASVs assigned to the *Bordetella* genus were present. *T. pyogenes, S. aureus,* and *K. pneumoniae* were rarely detected. We observed a peak of *T. pyogenes* in farm NLD1 samples containing a relative abundance >0.1*%* for a period from week 3 till week 8 (Fig. 4). *K. pneumoniae* was detected in the three German farms and in farm IRL3.

**TABLE 3:** Presence of bacterial pathogens in the 16S rRNA data set

Species	Prevalence in samples (*N* = 813)	Average relative abundance when detected	Average relative abundance overall	Maximum relative abundance in a sample
*Actinobacillus pleuropneumoniae*	136	1.96%	0.33%	56.43%
*Actinobacillus suis*	ND^*a*^	–	–	–
*Bordetella bronchiseptica*	ND^*a*^	–	–	–
*Glaesserella parasuis*	500	6.20%	3.81%	47.00%
*Klebsiella pneumoniae*	69	0.87%	0.07%	6.69%
*Mannheimia varigena*	365	7.50%	3.40%	63.71%
*Mycoplasma hyopneumoniae*	ND^*a*^	–	–	–
*Mycoplasma hyorhinis*	196	2.12%	0.51%	51.45%
*Pasteurella multocida*	84	1.02%	0.11%	17.12%
*Staphylococcus aureus*	11	0.29%	< 0.01%	0.69%
*Streptococcus suis*	804	3.15%	3.11%	25.43%
*Trueperella pyogenes*	55	0.29%	0.02%	3.20%

^*a*^ These species have not been detected in the sequencing data.

To investigate whether there were temporal trends in the presence of pathogens, we plotted the relative abundance of the pathogens investigated for 16S rRNA (Fig. 4) and *tuf* (Fig. S5) by farm. As expected from the high prevalence of *S. suis* (Table 2), this taxon was consistently detected across the studied farms. *A. pleuropneumoniae* was mostly detected in the first week, except for farm IRL3, where it increased in relative abundance after week 2. *G. parasuis* was detected throughout the sampling period, generally with a higher relative abundance after week 2 (except for farm GER3, where it was only detected in the first week).

We observed different profiles of pathogen presence between farms. For example, in farms GER1 and IRL1, hardly any *M. hyorhinis* or *P. multocida* were detected. In contrast, co-occurrence of these species was observed on farms IRL2, IRL3, and NLD1. *M. hyorhinis* was prevalent in five of the nine farms and mostly after week 2.

### Co- and anti-correlation of bacterial species in the piglet nasal microbiome

Positive and negative associations between the top ~125 taxa at species level for 16S and top ~50 taxa at species level for *tuf* for were determined using SparCC per country after removing species with low prevalence. The selected taxa explained an average of 94% of the relative abundance in the 16S and 99% in the *tuf* data set. Subsequently, a selection of species, with a SparCC correlation index smaller than −0.2 and larger than 0.2 (cutoffs were chosen to reduce the number of species and edges between species) per country, was included for clustering using a Markov cluster algorithm (MCL). We observed seven distinct co-abundance groups for 16S rRNA and two *tuf*-CAGs. 16S-CAG1 was the largest with 15 members, followed by 16S-CAG2 with 13 members. We further established two *tuf*-CAGs of similar size (seven and eight taxa) (Table S1).

**Fig 5:**
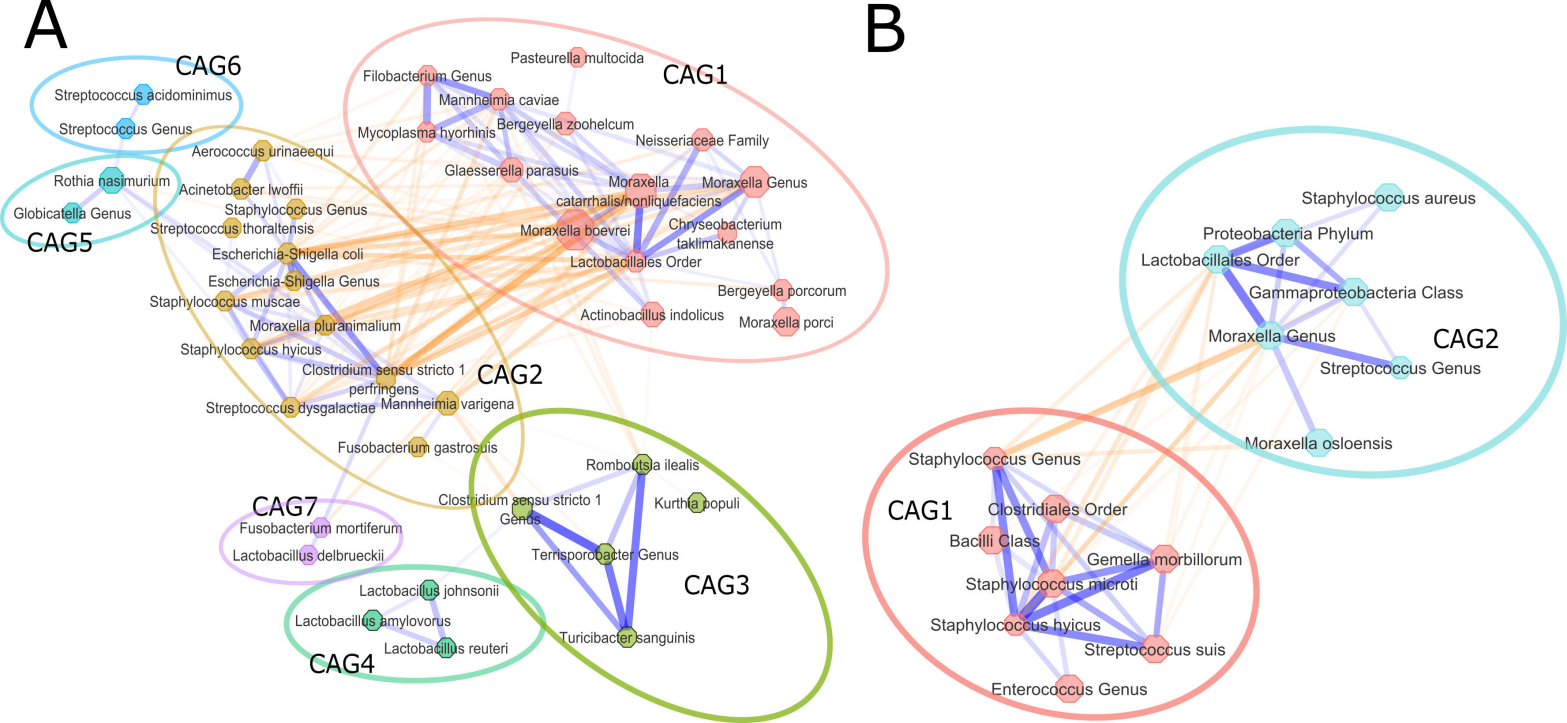
Network of co- and anti-correlating species for 16S rRNA (**A**) and *tuf* (**B**). Line color orange indicates negative association, and blue indicates positive association. Line thickness corresponds with a higher average correlation index (*R*^2^). The larger node size indicates the greater average relative abundance over the whole data set. Species nodes are colored and circled according to their CAGs.

The SparCC-inferred interactions of the species were visualized in a network (Fig. 5). Between the members of 16S-CAG1 and 16S-CAG2, exclusively negative interactions were observed. Members of 16S-CAG3 and 16S-CAG4 shared positive interactions between them and negative interactions with members of the other 16S-CAGs. The smaller 16S-CAG5, 16S-CAG6, and 16S-CAG7 had positive associations with members of 16S-CAG2.

### Temporal succession of taxa could underlie the co-abundance grouping

**Fig 6:**
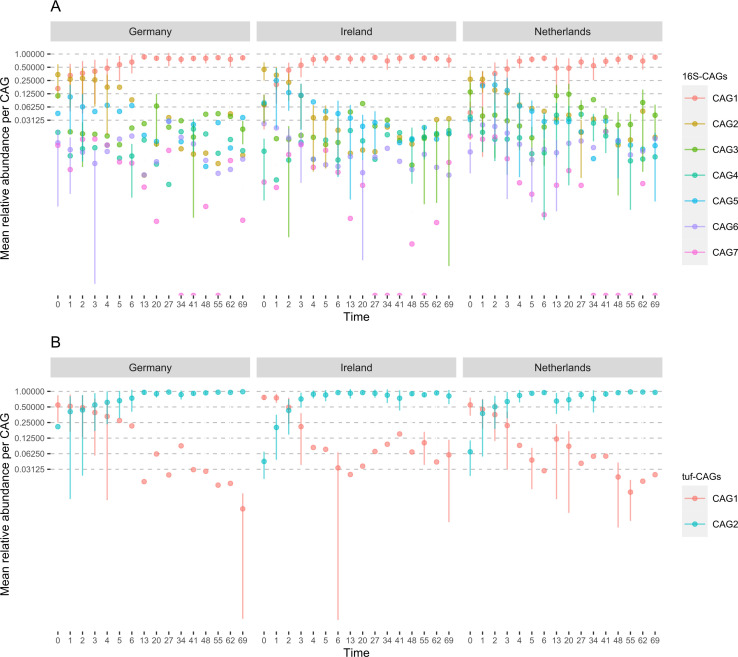
Average relative abundances of the seven 16S-CAGs (**A**) and two *tuf*-CAGs (**B**) by time, per country; error bars represent the standard deviation in relative abundance between samples.

Time explained most variability in microbial composition. To observe time effects on CAGs, the average of the summed relative abundances per CAG (Fig. 6) was plotted over time. In all three countries, similar trends were observed; 16S-CAG1 increased in relative abundance from day 1 and remained dominant up to day 70 of life. Inversely, the relative abundance of 16S-CAG2 was highest at birth and decreased from the first day, with some fluctuations in relative abundance later in life. Similarly, *tuf*-CAG1 was succeeded by *tuf*-CAG2 after day 1, a trend that was again evident in all three countries. 16S-CAG3 had a minor peak around week 2 of life (2 weeks prior to weaning). This effect was more evident in the unaveraged plot of 16S-CAG3 (Fig. S6). The 16S-CAGs other than the 16S-CAGs 1 and 2 had a generally low relative abundance.

### Candidate probiotic species that are anti-correlated with respiratory pathogens

We utilized the SparCC analysis of the 16S rRNA and *tuf* data sets to infer taxa negatively associated with airway pathogens over all three countries, displayed in Table 4. Porcine pathogens of 16S-CAG1 (*G. parasuis*, *M. hyorhinis*, and *P. multocida*) showed inverse correlations with members of 16S-CAG2, 16S-CAG3, and 16S-CAG5. *M. varigena*, a pathogen of 16S-CAG2, was negatively correlated with members of 16S-CAG1, 16S-CAG3, and 16S-CAG4 (Table 4). These anti-correlating taxa could be explored for probiotic potential.

**TABLE 4:** Significant anti-correlating taxa with common airway pathogens (dark grey)^*a*^

Pathogen	Taxon anti-correlated	Correlation	Data set	CAG	Pathogen	Taxon anti-correlated	Correlation	Data set	CAG
*G. parasuis*				**CAG1**	*M. hyorhinis*				**CAG1**
	*Clostridium sensu stricto 1 perfringens*	−0.35	16S	CAG2		*Clostridium sensu stricto 1 perfringens*	−0.33	16S	CAG2
	*Streptococcus dysgalactiae*	−0.30	16S	CAG2		*Escherichia-Shigella coli*	−0.30	16S	CAG2
	*Romboutsia ilealis*	−0.30	16S	CAG3		*Romboutsia ilealis*	−0.28	16S	CAG3
	*Turicibacter sanguinis*	−0.28	16S	CAG3		*Aerococcus urinaeequi*	−0.28	16S	CAG2
	*Staphylococcus hyicus*	−0.27	16S	CAG2		*Acinetobacter lwoffii*	−0.26	16S	CAG2
	*M. varigena*	−0.26	16S	CAG2		*Staphylococcus hyicus*	−0.25	16S	CAG2
	*Acinetobacter lwoffii*	−0.26	16S	CAG2		*Streptococcus dysgalactiae*	−0.25	16S	CAG2
	*Rothia nasimurium*	−0.26	16S	CAG5		*Moraxella pluranimalium*	−0.25	16S	CAG2
	*Aerococcus urinaeequi*	−0.26	16S	CAG2		*Turicibacter sanguinis*	−0.23	16S	CAG3
	*Moraxella pluranimalium*	−0.24	16S	CAG2		*Staphylococcus muscae*	−0.20	16S	CAG2
	*Escherichia-Shigella coli*	−0.23	16S	CAG2					
	*Acinetobacter* genus	−0.23	16S	–	*P. multocida*				**CAG1**
	*Staphylococcus muscae*	−0.22	16S	CAG2		*Moraxella pluranimalium*	−0.21	16S	CAG2
						*Clostridium sensu stricto 1 perfringens*	−0.20	16S	CAG2
*M. varigena*				**CAG2**					
	*Clostridium sensu stricto 1* Genus	−0.40	16S	CAG3	*S. aureus*			*tuf*	**CAG2**
	*Moraxella catarrhalis/nonliquefaciens*	−0.34	16S	CAG1		Bacilli class	−0.23	*tuf*	
	Lactobacillales order	−0.31	16S	CAG1					
	*Terrisporobacter* genus	−0.27	16S	CAG3	*S. suis*			*tuf*	**CAG1**
	*G. parasuis*	−0.26	16S	CAG1		Gammaproteobacteria class	−0.30	*tuf*	CAG2
	*Phascolarctobacterium succinatutens*	−0.21	16S	–		Lactobacillales order	−0.28	*tuf*	CAG2
	*Lactobacillus reuteri*	−0.21	16S	CAG4		*Moraxella* genus	−0.28	*tuf*	CAG2
	*Mannheimia caviae*	−0.20	16S	CAG1		*Moraxella osloensis*	−0.26	*tuf*	CAG2

^*a*^ The anti-correlation is based on the average SparCC *r* value over Germany, Ireland, and the Netherlands. All taxa displayed negative correlations (*P* < 0.0001) with an average *r* <−0.2 over the three countries.

## DISCUSSION

The development of the piglet nasal microbiome has implications for health and disease. However, its longitudinal development is understudied, and most microbiome studies lack taxonomic resolution (6). We obtained longitudinal data of the porcine nasal microbiota from birth to day 70 of life, from three farms across three different countries, using a combination of 16S rRNA and *tuf* gene amplicon sequencing achieving phylogenetic resolution up to species level. Using *tuf* sequencing, the species resolution of Firmicutes (35, 36) in the piglet nasal microbiome improved. This allowed us to describe CAGs of bacterial species that are commonly found together and consistently present in piglets in the studied countries.

### Piglet age and the farm shape the nasal microbiota

Time (age of the piglet) explained 19.4% of the (16S rRNA) variation in nasal microbiome composition between samples. The fact that the microbiome changes with time has been previously observed in human (37) and animal (38–40) studies, where a more stable microbiome has been observed in adulthood (37, 41). We observed a notable drop in alpha diversity during the first week, followed by a subsequent increase in the next week. This change in diversity was also reflected in the beta diversity. In other studies, changes in alpha diversity at T = 7 have been observed in relationship with antibiotic treatment of the sow or piglets (19) (in our study, no antibiotics were used). The authors hypothesized that their observed increase in alpha diversity was caused by dysbiosis and associated influx of environmental taxa.

Weaning is an impactful life event for piglets. This is reflected in a small but significant effect in beta diversity between samples before and after weaning. However, alpha diversity did not show a significant effect between the same timepoints. We observed that the alpha diversity stabilized after weaning and did not show significant differences. We observed a smaller difference between later timepoints than between earlier timepoints in the beta diversity analysis, which we visualized in the PCA plots (Fig. 3). This post-weaning stabilization has previously been described (20) and suggests maturation from juvenile to a more “adult” microbiome in response to the changing factors around weaning. After age, farm had the largest effect on beta diversity, followed by sow (litter) and the country of origin (Table 2). A farm effect (11) and a litter effect on the piglet-tonsil microbiota (42) have previously been described. Nonetheless, remarkable congruence in the developing microbiome was observed for all piglets across all countries. Homogeneity in environmental conditions such as gaseous ammonia (18) or using vaccination (43) and similar genetics (despite pig lineage differences between farms in this study) in commercial pig farming might help explain this consistency. To determine drivers behind the differences between farms, countries, and/or litters that affect nasal microbiome development in greater detail, a study design with more farms and farm data would be needed.

### The vaginal and fecal microbiome “seed” the nasal microbiome

Previously, we observed that nasal samples taken from piglets directly after birth contained many species associated with the gastrointestinal tract (7). An effect of natural birth on the piglets’ microbiome development is supported by the different development of the piglet’s (gut) microbiome in cesarean-section-derived pigs (44). Likely because for naturally born piglets, the first contact with the nonsterile environment, including the birth canal and maternal feces, is different. A maternal effect is also suggested in studies where the sow microbiome and the sow’s parity influenced the piglet tonsillar microbiome directly post-partum. As was seen in the vaginal microbiota of high-parity sows consisting of taxa as Pasteurellaceae family, and the *Aerococcus, Clostridium,* and *Escherichia* genera. These taxa were subsequently more present in their piglets’ tonsil directly post-partum (45). Similarly, in our samples shortly after birth, we detected members of the genera *Clostridium* and *Escherichia*, taxa often associated with the gut (Fig. 2). *Clostridium* and *Escherichia* species clustered into 16S-CAG2 and 16S-CAG3 (16S-CAG3 almost completely consisted of Clostridiaceae). Both these CAGs were detected mostly in early life (CAG3 has an additional peak around week 2). The relative abundance of these gut-associated taxa and the higher Shannon alpha diversity (Fig. 1) indicate nasal colonization by vaginal and (the generally rich) fecal microbiota. A recent study shows that gut-associated microbes are present nasally and actively living there (46). These observations add further evidence that natural birth seeds the early colonizers of the piglet’s upper respiratory tract.

### Sow and farrowing pen conditions shape the starting microbiome

The samples from the first week after birth excluding the first timepoints (T = 2–6) likely consist of a microbiome that is driven by maternal or farrowing pen influences as the samples of these timepoints cluster together horizontally. Colostrum and the high piglet snout to sow skin/teat contact may shape the microbiota, as has been suggested by Obregon-Gutierrez and collaborators. Specifically, sow-skin contact was associated with presence of the genera *Rothia*, *Moraxella*, and *Enhydrobacter* (47). *Enhydrobacter*, a member of the Moraxallaceae family, was not observed in our study. However, we did observe high abundances of *Rothia*, albeit less abundant than *Moraxella* and *Mannheimia* species, mainly present during the first week of life. This is supported by our previous finding that *Rothia* was present at almost 50% relative abundance during the first week (7). *M. boevrei* was often the most prevalent species during this period, succeeded later in life by other *Moraxella* species, a finding consistent with other studies (12, 13, 48, 49). Interestingly, *Moraxella* has been observed as a common nasopharyngeal inhabitant in humans and animals and has been associated with opportunistic airway infections (10, 50–52), but also with breastfeeding, and microbiome stability (41).

### Abundant taxa detected in the nasal microbiota match previous studies

In alignment with our data (Fig. 3), Correa-Fiz et al. (12) identified genera, such as *Moraxella, Haemophilus, Enhydrobacter, Klebsiella, Oscillospira, Streptococcus, Lactobacillus, Weeksella, Prevotella,* and *Bacteriodes,* as key components of the core nasal microbiome at 3–4 weeks of life (12). The discrepancy between *Haemophilus* and *Glaesserella* can be explained by a name change of *H. parasuis* to *G. parasuis*. The most abundant genera in their 2019 study (48) again correlate with our current findings. The porcine nasal microbiome samples studied by Strube et al. (32) had higher *Streptococcus*, *Rothia, Moraxella,* and *Globicatella* and lower *Facklamia* and *Aerococcus* abundances. Again, we observed these genera, except *Facklamia* and *Globicatella,* as our most abundant taxa. The most abundant genera in our study are also in direct concordance with the genera present in the pig upper respiratory microbiome, as found at three timepoints by Rampelotto et al. (13) at the end of weaning, at the end of the nursery phase (T = 71), and at finishing (T = 373) (13).

### Co-abundance analysis identifies two major CAGs in competition

In both 16S rRNA and *tuf* data sets, co-abundant species grouped across countries. Previously, we described CAGs from 16S rRNA and *tuf* nasal swab data sets in one Dutch farm with temporal trends and associations with LA-MRSA (7). Here, we describe seven 16S-CAGs across multiple farms and countries. There was overlap between 16S-CAG6 and 16S-CAG8 of the previous study and 16S-CAG1 and 16S-CAG3 of the current study. For example, in previous 16S-CAG8 and current 16S-CAG3, we observe *Clostridium* species being present 2 weeks pre-weaning.

The interaction network depicts that the members of the two largest 16S-CAGs are negatively associated with each other (Fig. 5A). The same holds true for the two *tuf*-CAGs we defined (Fig. 5B). These negative associations have a temporal component. Members of 16S-CAG1 and *tuf*-CAG2 are most present in the later timepoints, indicating that these CAGs are part of the microbiome of older piglets. We argue that there is a group of genera colonizing the nasal microbiome at birth and early in life (e.g., the species in 16S-CAG2). This group is later succeeded by other taxa (the species in 16S-CAG1) potentially due to microbe competition, changing maternal contact, piglet development, or by altered piglet behavior and changes in its diet (e.g., weaning or early life solid feed introduction).

### Several taxa were negatively associated with respiratory pathogens

We observed widespread presence of opportunistic pathogens, with low relative abundances, and observed farm-specific pathogen trends. Several taxa were negatively associated with pathogens (Table 4). For example, some farms showed *M. hyorhinis* in co-occurrence with *P. multocida* after week 2. This is in concordance with clinical signs of *M. hyorhinis* infections arising around weeks 3–10 in affected pigs (30) and matches previous descriptions of secondary infections of *M. hyorhinis*, *P. multocida,* and *S. suis* after viral infection as part of the PRDC complex (53). However, piglets in our study did not show clinical signs. This absence of signs is consistent with studies that detected bacteria commonly associated with disease without causing disease (21, 54). Therefore, these species could be considered as opportunistic pathogens or pathobionts (55). One thing to note is that taxonomy assigned to amplicon sequencing does not account for within-species heterogenicity nor can it detect the presence of virulence genes. Therefore, we were not able to discriminate between commensal and virulent strains that were present in the nasal microbiome of the sampled pigs.

Before using the current findings to develop competitive exclusion against respiratory pathogens, additional experimental evidence is warranted. The amplicon sequencing used in the current study resulted in compositional data, which are not ideal for quantitative analysis (56). Absence of taxa in the sequence data does not necessarily indicate a true absence of the bacterium (7). Furthermore, methodological differences can cause effect on the outcome of a sequencing effort and may cause differences between studies (57). Quantitative and sensitive methods such as qPCR are needed to confirm negative correlations between potential probiotic taxa and opportunistic pathogens. Additionally, interactions identified in our *in silico* analyses should be tested by isolating identified strains and testing them in *in vitro* and *in vivo* models.

### Conclusion

This study extends the understanding of the longitudinal development of the nasal microbiome of 54 piglets between birth and day 70 of life. The nasal microbiota displayed similar trends in (alpha- and beta-) diversity and relatively abundant taxa over three geographically distinct regions, in different farms, and different litters. We conclude that the development of the piglet nasal microbiome is strongly influenced by the age of the pig regardless of the farm. CAGs of bacterial species, with distinct temporal trends in all three countries, showed interactions between commensal nasal species and respiratory pathogens. These data can be used to inform and identify probiotic strains to control opportunistic bacterial airway pathogens. Such a pathogen reduction strategy would be valuable in the fight against AMR.

## MATERIALS AND METHODS

### Farms and animal management

Nine conventional farms were studied in three European countries [Ireland (*n* = 3), Germany (*n* = 3), and the Netherlands (*n* = 3)]. Farmers gave informed consent to participate in the study, and all data were anonymized before analysis. At birth, piglets from four litters on each of the three farms in the three countries were selected for nasal swabbing. Only piglets from sows which had not been treated with antimicrobials were selected, and piglets from litters where antimicrobials were administered during the study period were excluded. Farm-specific details can be found in the questionnaire results (Table S2).

### Nasal swab sampling

Piglets were nasal swabbed at 0, 1, 2, 3, 4, 5, 6, 13, 20, 27, 34, 41, 48, 55, 62, and 69 days after birth. Nasal swabs were taken from 54 piglets born to 27 sows across 9 farms over 3 countries (*N* = 864). For samples taken in week 1, aluminum-wire swabs with a rayon tip (Copan, 160C) were used. Swabs from later timepoints (T = 14–69) were taken using plastic swabs with a rayon tip (Copan, 155C). The swabs were kept refrigerated and were processed within 24 hours post-sampling. The swab tips were cut into 2-mL Eppendorf tubes and submerged in 600-µL lysis buffer BL (LGC genomics, Berlin, Germany) and stored at −20°C until processing.

### DNA extraction

DNA from nasal swabs was extracted, from piglets with a completed timeseries (*n* = 16) [for Dutch farm NLD3 (*n* = 9)] resulting into a total of 813 samples, using a modified LGC mag kit protocol (LGC genomics, Berlin, Germany) adapted from Wylie et al. (58). The samples were homogenized and lysed by beat beating with 0.2-mm zirconia beads and 500-µL molecular grade TE-saturated phenol (ThermoFisher, Bleiswijk, the Netherlands). After spinning, the aqueous phase was transferred to 1,000 µL binding buffer and 10 µL magnetic beads in a round bottom molecular grade 2.2 mL 96-deep-well plate (VWR international, Amsterdam, the Netherlands). The binding and washing steps were performed in 96-well plates according to the manufacturer’s specification, and the eluted DNA was stored at −20°C. Empty lysis buffer was used as negative controls for DNA extraction, qPCR, and sequencing.

### Quantification of 16S rRNA by real-time PCR to normalize sequencing input

Sample bacterial DNA was estimated using a 16S rRNA qPCR to normalize the sequencing library preparation. Reactions were performed on the LightCycler 480 platform (Roche Diagnostics, Almere, The Netherlands). Reaction mixtures consisted of 1 µL nasal swab DNA, 7 µL molecular grade water, 1 µL primer 355F (5*'* ACTCCTACGGGAGGCAGC 3*'*) at 10 µM, 1 µL primer 556R (5*'* CTTTACGCCCARTRAWTCCG 3*'*) at 10 µM, and 10 µL SYBR Green Master Mix (Bio-Rad, Veenendaal, The Netherlands). The DNA extraction estimated an average rRNA yield corresponding to 5 × 10^8^ bacterial cells/mL.

### 16S rRNA and *tuf* gene sequencing

The V3-V4 region of the 16S rRNA gene was amplified using the primers 341F (5′-TCGTCGGCAGCGTCAGATGTGTATAAGAGACAGC

CTACGGGNGGCWGCAG-3′) and 805R (5′-GTCTCGTGGGCTCGGAGATGTGTATAAGA GACAGGACTACHVGGGTATCTAATCC-3′) (Eurofins Genomics, Germany). The *tuf* gene was amplified using the primers tuf-F (5′-GCCAGTTGAGGACGTATTCT-3′) and tuf-R (5′-CCATTTCAGTACCTTCTGGTAA-3′) (36). Both primer sets were used at a concentration of 0.2 µM with Phusion High-Fidelity DNA polymerase (Thermo Scientific, USA). According to Illumina (San Diego, CA, USA) recommendations, the following PCR conditions were used: an initial denaturation step at 98°C for 30 s, followed by 25 cycles of denaturation at 98°C for 10 s, annealing at 55°C for 15 s, extension at 72°C for 20 s, and a final extension step at 72°C for 5 min, followed by cooling to 4°C. The amplicons were visualized on a 1% agarose gel to confirm amplicon lengths of approximately 550 bp for the 16S rRNA gene and 400 bp for the *tuf* gene. The amplicons were purified using Agencourt AMPure XP magnetic beads (Beckman-Coulter, USA) and eluted in 50 µL EB Buffer (Qiagen), after which Illumina barcode sequences were added using the Nextera XT v2 Index Primer Kit. The index PCR conditions were as follows: 98°C for 30 s, followed by eight cycles of denaturation at 98°C for 10 s, 55°C for 15 s, 72°C for 20 s, and a final 72°C for 5 min, followed by cooling to 4°C. Another round of purification with Agencourt AMPure XP magnetic beads was performed, and the final amplicons with indices were eluted in 28  µL EB Buffer (Qiagen). Sample concentration was measured using the Qubit High-Sensitivity Double-Stranded DNA Assay Kit (Thermo Scientific) on a Qubit 3 Fluorometer. For library pooling, 30 ng DNA from each sample was combined to create a randomized pooled library that was sequenced at the Teagasc NGS sequencing facility (Teagasc Moorepark, Fermoy, Co. Cork, Ireland) on an Illumina MiSeq, generating 2  ×  300  bp paired-end reads.

### Amplicon sequencing read pre-processing

The check of read quality and the pipeline to infer ribosomal sequence variants (ASVs) using DADA2 v1.20 was performed as described by Patel et al. (7) with slight modifications considering the following parameters: truncLen = c(220,220), trimLeft = c (17, 21), maxEE = c (2, 2), truncQ = c (2, 2), maxN = 0, rm.phix = TRUE for 16S rRNA and truncLen = c(240,200), trimLeft = c (20, 22), maxEE = c (2, 2), truncQ = c (2, 2), maxN = 0, rm.phix = TRUE for the *tuf* gene. In the taxonomy assignment step, the bootstrap confidence threshold was mutated to 80%.

### Amplicon sequencing microbiome analysis

Data sets were formatted into two phyloseq objects (*tuf* and 16S rRNA) for diversity analyses, visualization, and statistics. RStudio for windows (2023.03.1+446 “Cherry Blossom” Release) with R version 4.1.3 (10 March 2022) was used to perform all analyses. Analyses in R were performed using the “microViz” (0.10.6) (59), “microbiome” (1.16.0), “tidyverse” (1.3.2), “CoDaSeq” (0.99.6), “phyloseqCompanion” (1.0), “vegan” (2.6–4), and “phyloseq” (1.38.0) packages. The full R analysis, including all loaded libraries (utilitarian libraries), is appended as a Quarto-document (.qmd).

### Alpha diversity calculations

Alpha diversity measures were estimated and visualized using the “phyloseq” R-package. To correct for varying sequence depth, all samples were rarefied to 5,034 reads for the 16S rRNA data set (minimum read count of the 16S rRNA data set) and 6,091 reads for the *tuf* data set (minimum read count of the *tuf* data set). Shannon alpha diversity was visualized using the “plot_richness()” function. *t*-Tests to compare timepoint alpha diversity were performed using the base R Welch two sample *t*-test at an alpha of 0.05.

### Beta diversity calculations

Aitchison distances were calculated from centered log-ratio) transformed, unfiltered, and unrarefied data sets, which had zeroes imputed with half the minimum observed value for each taxon at species level, using the “tax_transform()” function. When taxonomy was not assignable to species level, the microViz “tax_fix()” function was used to impute the highest possible taxonomic resolution (E.G. *Staphylococcus* species). Samples were ordinated using the “ord_calc()” function using method “PCA.” The ordinations were visualized using the “ord_plot()” function. Top 15 ordination-driving species were visualized by using the “ord_plot()” setting; “plot_taxa = 1:15.” All three functions are from the “microViz” R-package.

### PERMANOVA analysis on beta diversity

PERMANOVA analysis was performed on the ordinations asmentioned in the result section above using the “adonis2()” function from the “Vegan” R-package. Use the model formula: “Country/Farm/Identifier_SOW/Identifier_pig +Time + Sex” to consider the nested study design. We used 5,000 permutations. Pairwise comparisons were made using the “pairwise.adonis2()” function from the “PairwiseAdonis” R package with an alpha of 0.05.

### Visualization of microbial composition

Composition bar plots were generated by merging relative abundance data from all farms by timepoint for each country. Unrarefied data sets were used to generate the composition bar plots. Bar plots were generated using the “comp_barplot()” function, and heatmaps were generated using the “cor_heatmap()” function both from the “microViz” R-package, setting tax_level to “species,” and n_taxa to 25. Figures were optimized using functions from the “ggplot2” R package.

### SparCC correlation

ASVs were agglomerated to species level to generalize species-level effects. Positive and negative associations based on the abundances of species with at least 50 reads in at least 5% of the samples in the 16S rRNA and *tuf* data set were inferred using SparCC (60). From each separate country, (anti)correlations with an *r* >0.2 or *r* <−0.2 were selected. Species displaying significant correlation with an average correlation of *r* >0.2 over all countries, where the interaction was found, were clustered into co-abundance groups using MCL (61) using default settings.

## Data Availability

STORMS files, scripts, metadata, and Phyloseq objects are available through ZENODO. Reads are available under accession number: PRJEB71383.

## SUPPLEMENTAL MATERIAL

Supplemental figures and tables msystems.00626-24-s0001.pdf Fig. S1-S6; Tables S1 and S2.

ASM does not own the copyrights to Supplemental Material that may be linked to, or accessed through, an article. The authors have granted ASM a non-exclusive, world-wide license to publish the Supplemental Material files. Please contact the corresponding author directly for reuse.
