# Supplementary material for: The developing pig respiratory microbiome harbors strains antagonistic to common respiratory pathogens
Source: mSystems. 2024 Sep 17;9(10):e00626-24. doi: 10.1128/msystems.00626-24 (PMC11494925; doi:10.1128/msystems.00626-24)
Supplement: Supplemental figures and tables — Fig. S1-S6; Tables S1 and S2. [file msystems.00626-24-s0001.pdf]

Figure S.1A Rarefaction curve of all piglets 16S rRNA samples pre rarefaction. This is a visualization of the increasing species count when rarefying through the sequences of each sample. Flattening curves indicate that sufficient read depth has been achieved to represent the ribosomal sequence variants in the sequenced 16S rRNA gene.

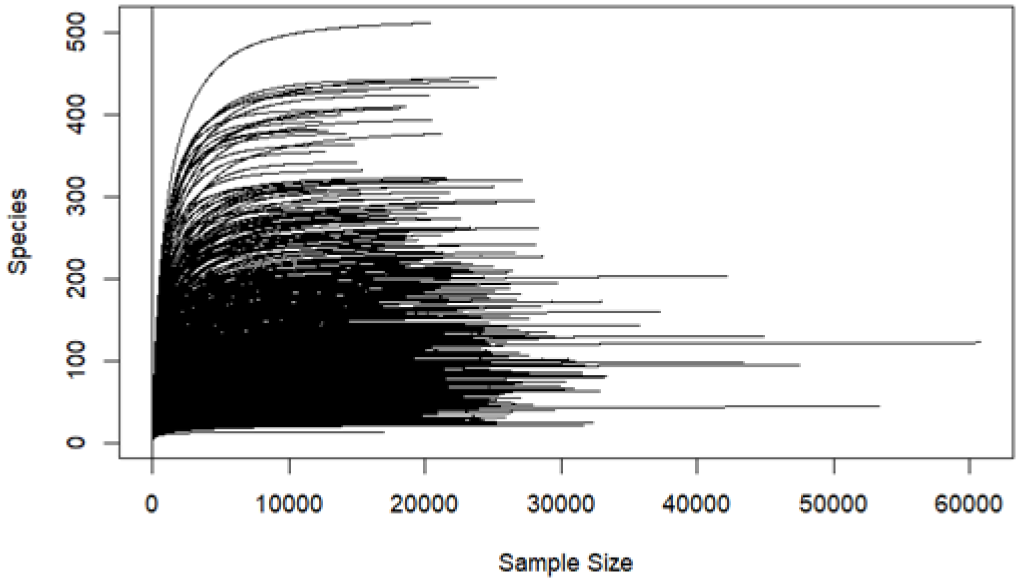

Figure S.1B Rarefaction curve of all piglets *tuf* samples pre rarefaction. This is a visualization of the increasing species count when rarefying through the sequences of each sample. Flattening curves indicate that sufficient read depth has been achieved to represent the *tuf* sequence variants in the sequenced *tuf* genes.

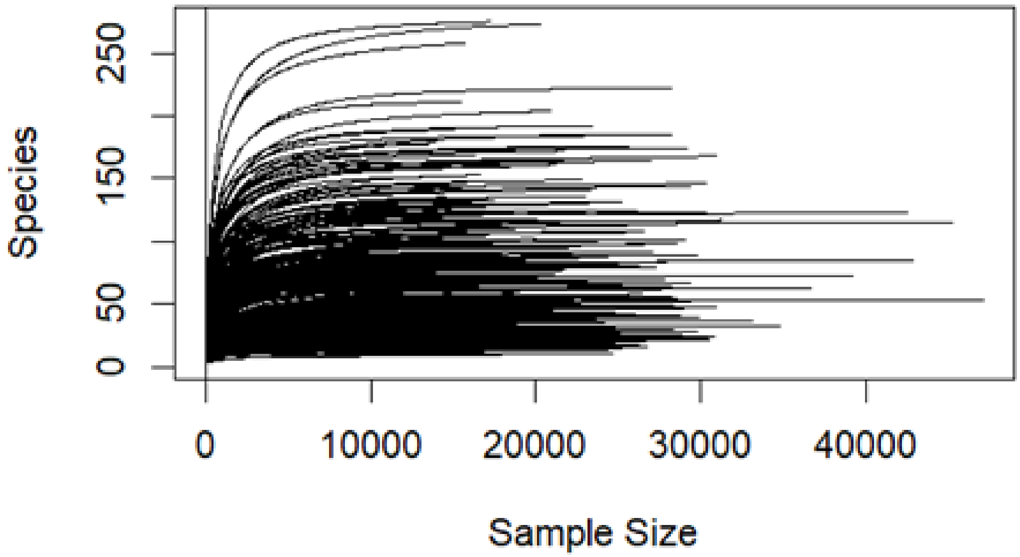

Figure S.2 A Distribution of read depth 16S rRNA gene sequencing **B** Distribution of read depth *tuf* sequencing.

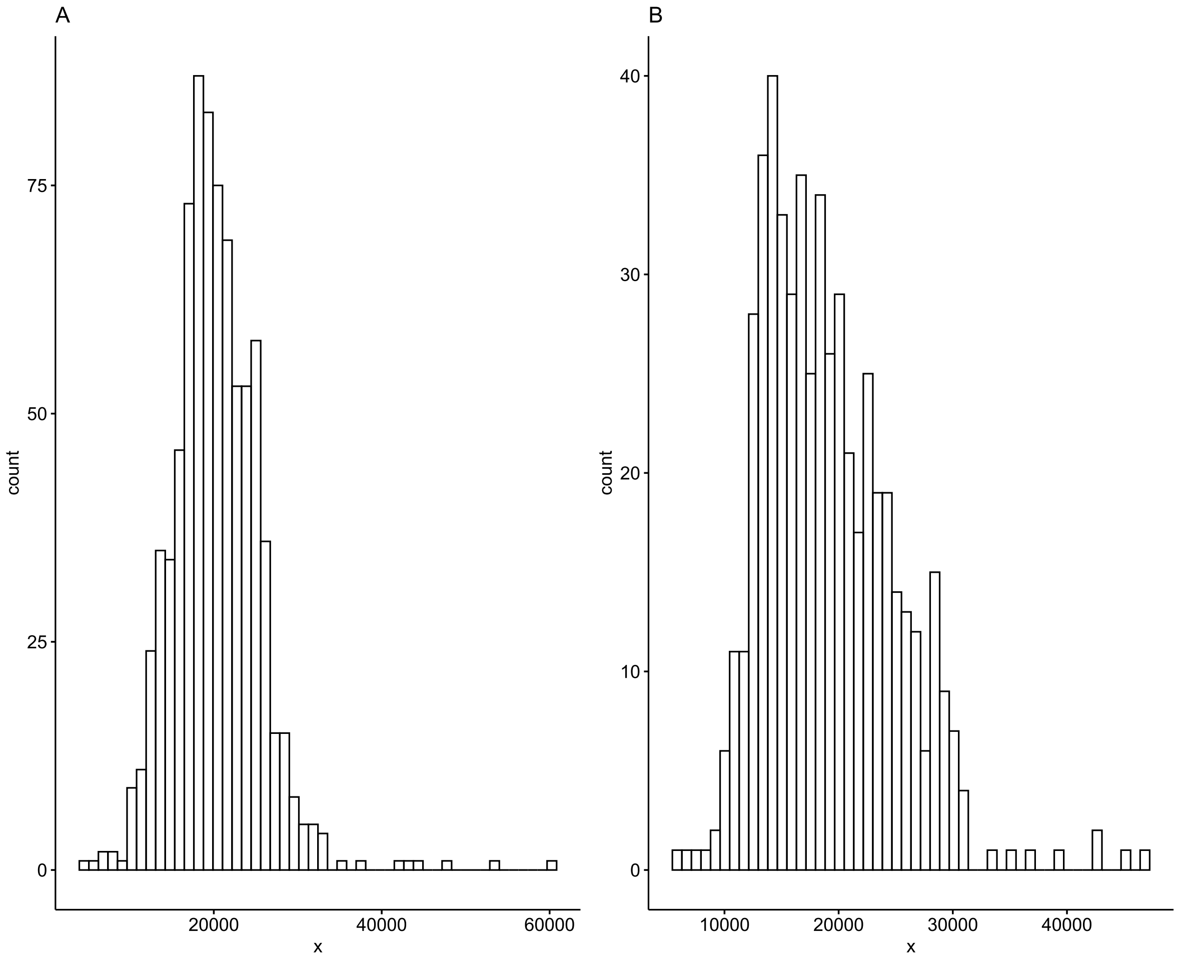

Figure S.3 Longitudinal changes in the piglet nasal community Shannon diversity measured for **A:** 16S rRNA and **B**: *tuf* gene amplicon sequencing. X-axis: sample time (days-categorical), timepoint 0 represents the piglets' day of birth. Y-axis: Shannon diversity index. Farms are presented by country, and color coded for identification. The dots represent the samples, lines display the predicted mean and the grey boundaries the standard error.

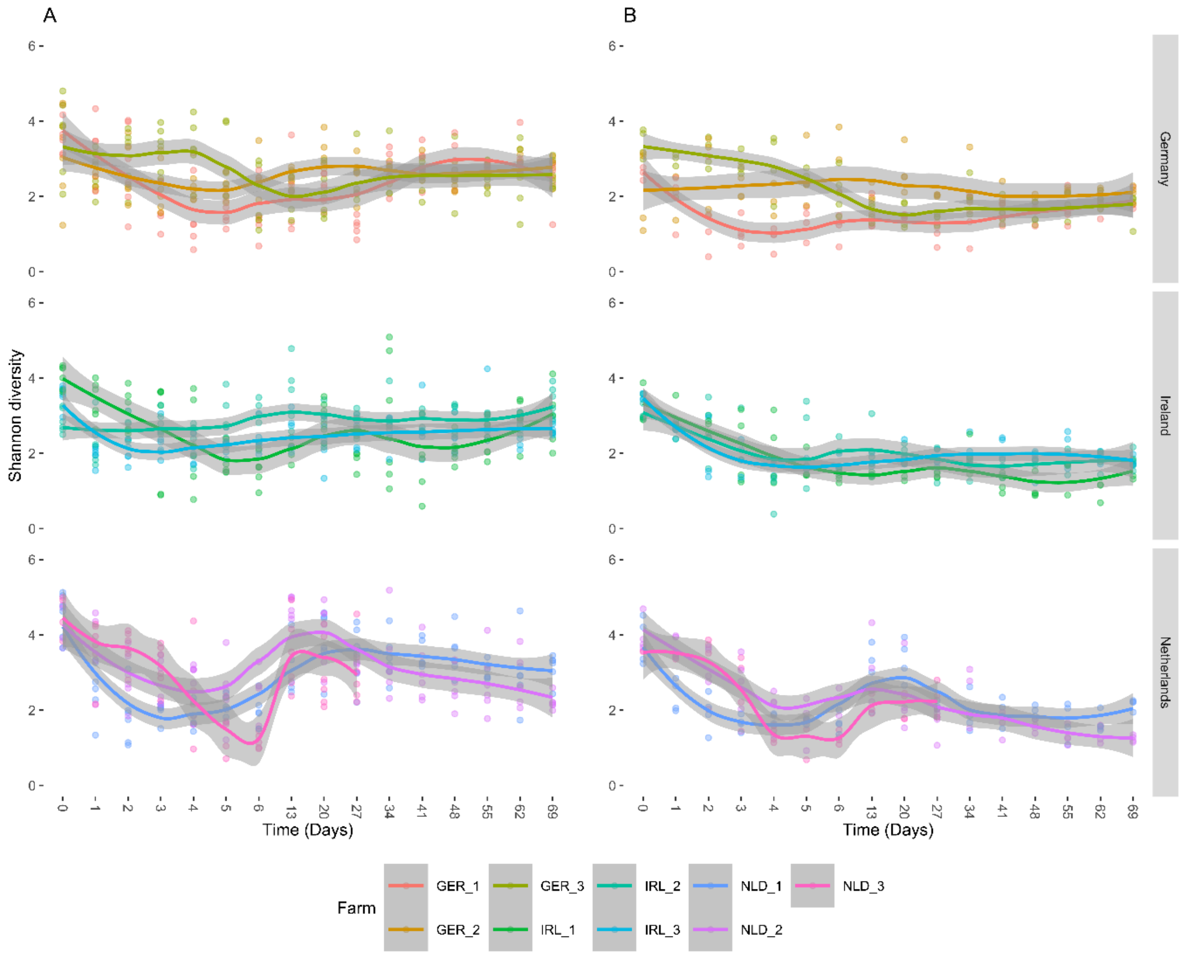

Figure S.4. A Top 25 species from the 16S rRNA dataset of all samples compressed into a one bar per timepoint per farm

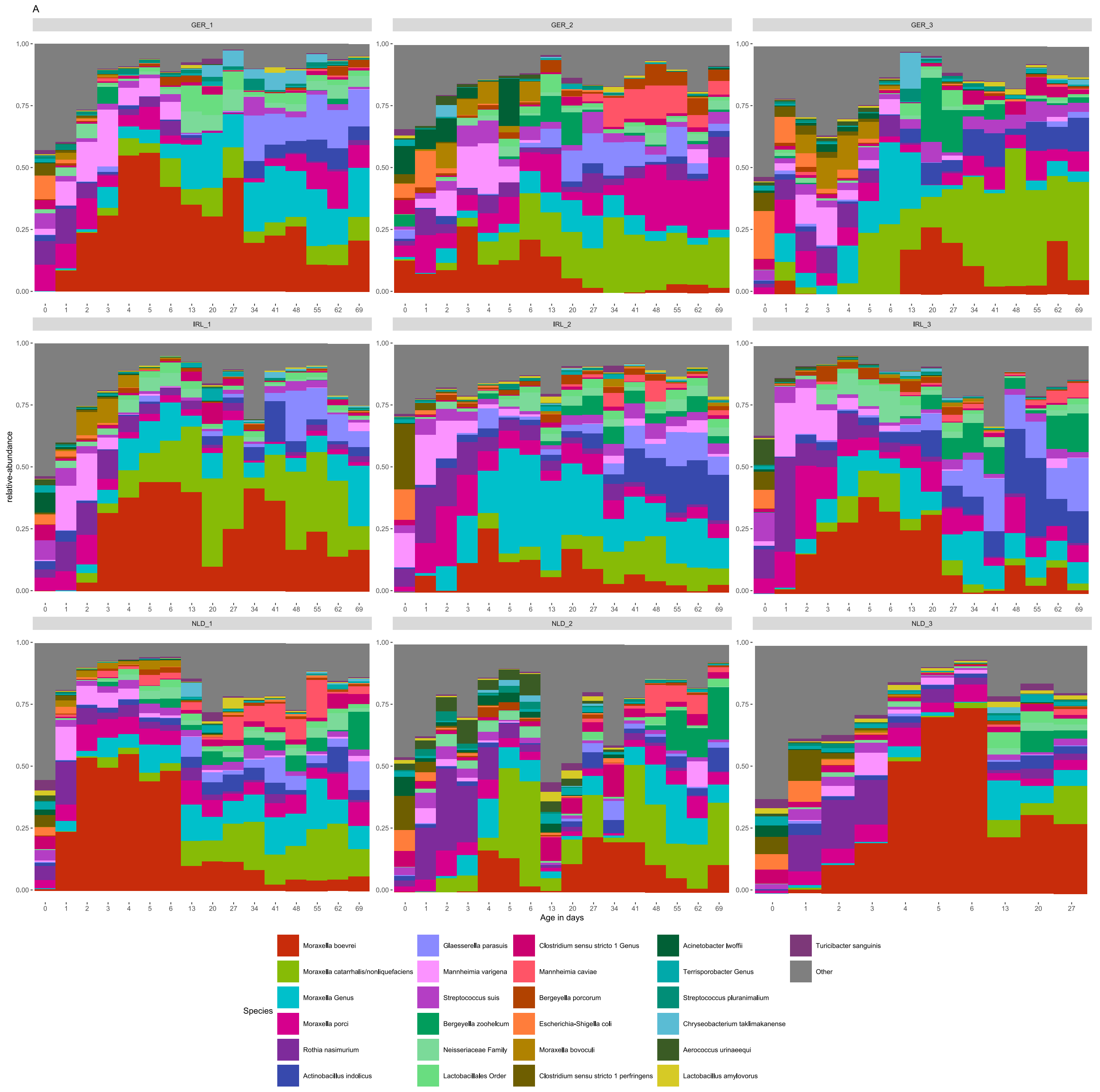

Figure S.4. **B** Top 25 species from the *tuf* dataset of all samples compressed into a one bar per timepoint per farm.

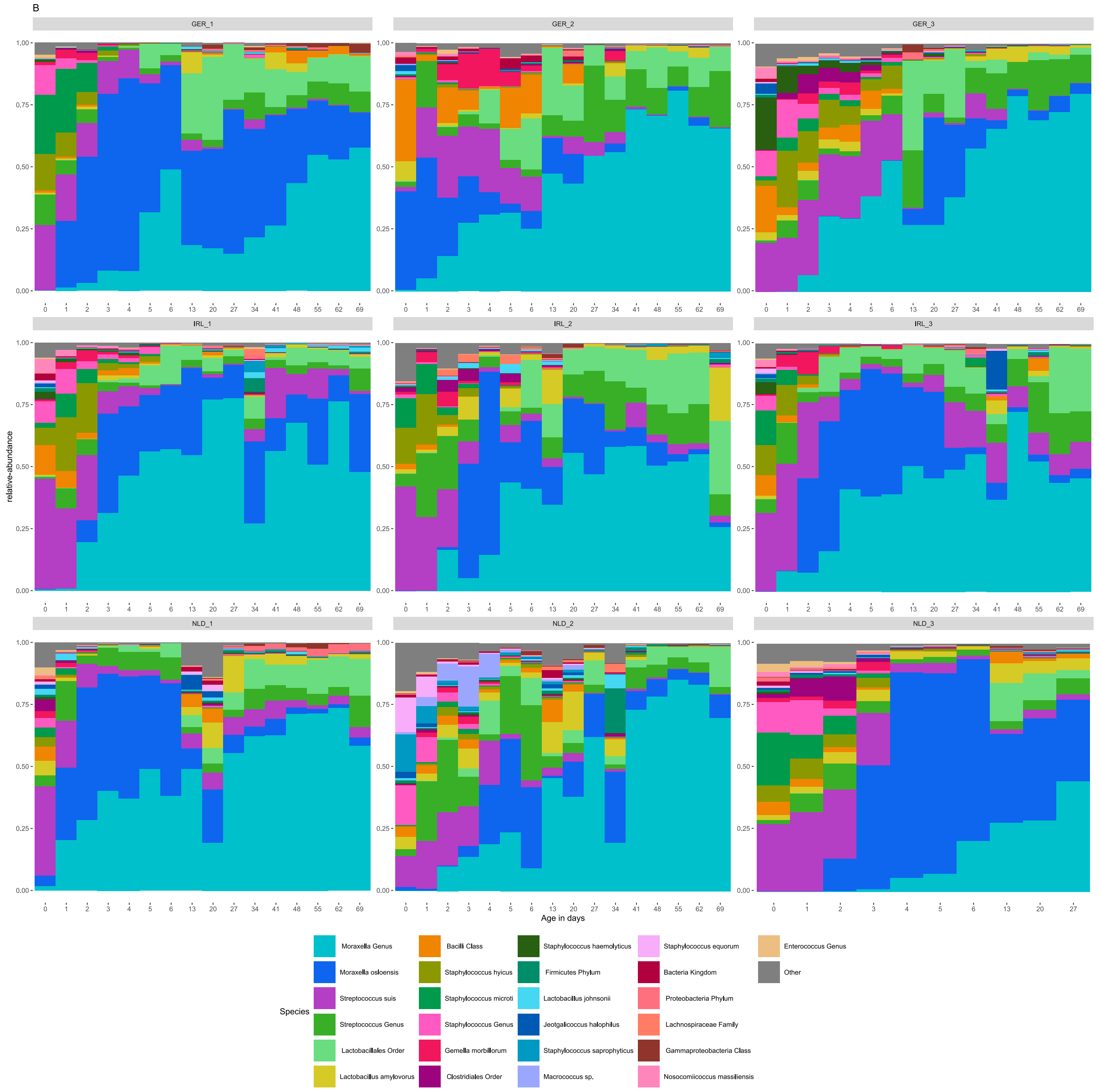

Figure S.5 *tuf* pathogens relative abundances per sample in time.

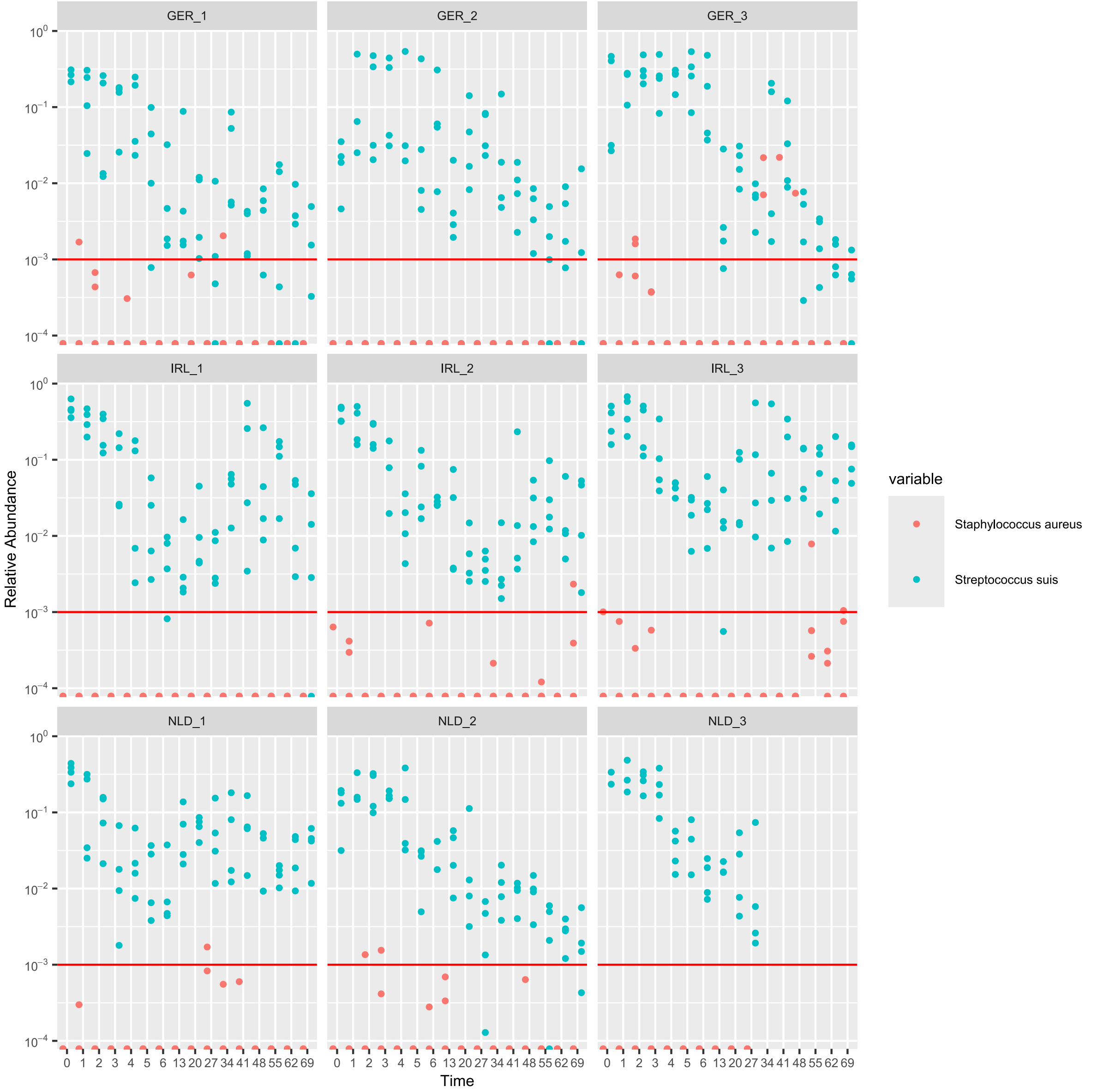

Figure S.6 Dotplot displaying relative abundances of 16S-CAG3 per sample. Relative abundance of 16S-CAG3 spikes around weaning (T27).

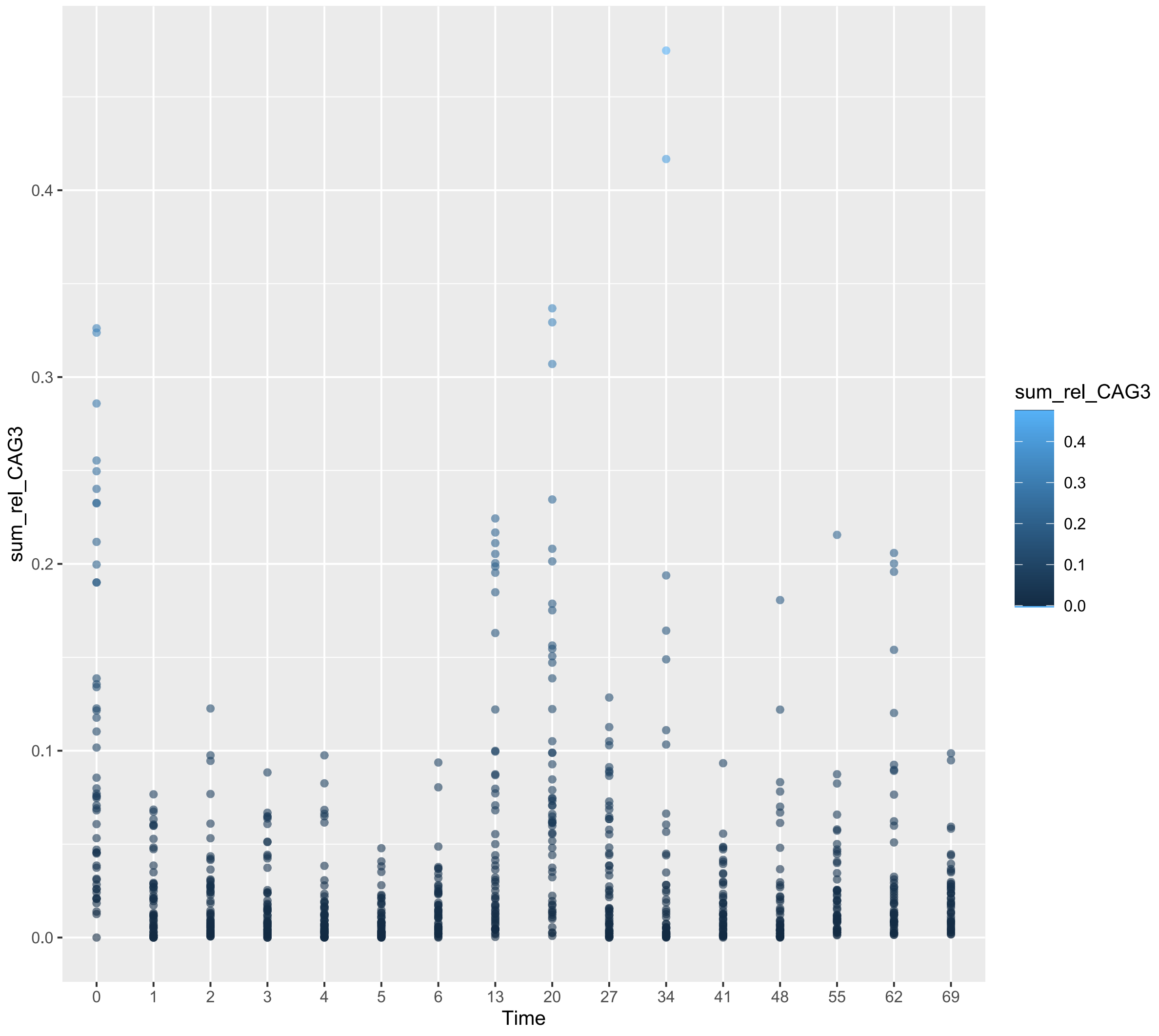

Table S.1. Co-abundance groups (CAGs) in the 16S rRNA and *tuf* dataset with an average co-correlation of r>0.2 over all three countries.

Species level Taxa	16S-CAG
Chryseobacterium taklimakanense	1
Neisseriaceae Family	1
Moraxella Genus	1
Glaesserella parasuis	1
Moraxella catarrhalis/nonliquefaciens	1
Bergeyella zoohelcum	1
Pasteurella multocida	1
Bergeyella porcorum	1
Lactobacillales Order	1
Actinobacillus indolicus	1
Mycoplasma hyorhinis	1
Moraxella boevrei	1
Mannheimia caviae	1
Filobacterium Genus	1
Moraxella porci	1

Species level Taxa	16S-CAG
Clostridium sensu stricto 1 perfringens	2
Streptococcus thoraltensis	2
Staphylococcus hyicus	2
Staphylococcus muscae	2
Mannheimia varigena	2
Staphylococcus Genus	2
Aerococcus urinaeequi	2
Acinetobacter lwoffii	2
Streptococcus dysgalactiae	2
Escherichia-Shigella coli	2
Fusobacterium gastrosuis	2
Moraxella pluranimalium	2
Escherichia-Shigella Genus	2

Species level Taxa	16S-CAG
Romboutsia ilealis	3
Kurthia populi	3
Clostridium sensu stricto 1 Genus	3
Turicibacter sanguinis	3
Terrisporobacter Genus	3
Lactobacillus johnsonii	4
Lactobacillus amylovorus	4
Lactobacillus reuteri	4
Rothia nasimurium	5
Globicatella Genus	5
Streptococcus Genus	6
Streptococcus acidominimus	6
Fusobacterium mortiferum	7
Lactobacillus delbrueckii	7

Species level taxon	*tuf*-CAG
Bacilli Class	1
Staphylococcus microti	1
Staphylococcus Genus	1
Staphylococcus hyicus	1
Enterococcus Genus	1
Streptococcus suis	1
Gemella morbillorum	1
Clostridiales Order	1

Species level Taxa	*tuf*-CAG
Gammaproteobacteria Class	2
Streptococcus Genus	2
Lactobacillales Order	2
Staphylococcus aureus	2
Moraxella Genus	2
Proteobacteria Phylum	2
Moraxella osloensis	2

Table S.2 Farm characteristics

Suppl. Table S2. Farm characteristics

Farms	NLD1	NLD2	NLD3	GER1	GER2	GER3	IRL1	IRL2	IRL3
The race of the sow?	tn70	tn 50	tn 20	Danen	BHZP	BHZP	Landrace x Large White	Landrace x Large White	Landrace x Large White
The race of the boar?	tn tempo320	tn Tempo320	tn tempo	Pietrain	Pietrain	Pietrain	Duroc	Duroc	Duroc (PIC®800)
Are piglets vaccinated?	yes	no	Yes	Yes	Yes	Yes	Yes	Yes	Yes
At what age?	3 weeks	-	week 3	3-4 weeks	3-4 weeks	3-4 weeks	Weaning (26-28 days old)	Weaning (21-29 days old)	Weaning (28 days old)
What vaccine?	Circo/myco plasma	-	IDAL: PRRS PCV2 Mycoplasma hyopneumoniae	Hyogene/Circoflex	Mycoflex/Circoflex	Suvaxyn (Circa + MH RTU)	PMWS/ PCV2	PCV and Mhyo	PCV and MHyo
What age/weight are the piglets weaned	27 days ~8-9kilo	4 weeks	27 days	4 Weeks	4 Weeks	4 Weeks	26-27 days (8 Kg)	31 days (8 Kg)	28 days
During farrowing is there a type of feed available beside the mother's milk?	yes	yes	yes	yes	yes	yes	yes	yes	yes
If yes, what kind of feed?	milk cups/dryfeed	milkformula	Milkformula, starter, weaningfeed	Prestarter	Prestarter	Prestarter	Starter at farrowing	Starter at farrowing	Starter at farrowing

Is there a floor cover (straw, sand, pellets)?	silverdust/chalk	sawdust or chopped straw	no	no	no	no	No	No	Paper
Are rooms cleaned between cycles	yes	yes	yes	Yes	yes	Yes	Yes	Yes	Yes
By which method?	topfoam sometimes heavy decontamination if performance drops	water,topfoam	megades schippers	Water	Water	Water	Soak, power-wash and desinfectant (hyperox)	Power-washing and desinfectant (interkokask)	Power-wash, dry-out and desinfectant
At what age are the piglets ear tagged?	early	-	day 3	Day 1	Day 1	Day 1	Depends	Only gilts for replacement	Only gilts for replacement
Are the piglets teeth polished (filed) ?	sometimes	no	no	When needed	When needed	When needed	Yes	Yes	Yes
Are the piglets tails cut?	yes	yes	yes	Yes	Yes	Yes	Yes	Yes	Yes
Are piglet boars neutered/castrated?	no	no	no	yes	yes	yes	no	no	no
